# Paranasal sinuses malignancies: A 12-year review of clinical characteristics

**DOI:** 10.4317/medoral.21170

**Published:** 2016-07-31

**Authors:** Seyed-Amir Danesh-Sani, Alireza Sarafraz, Mojtaba Chamani, Hossein Derakhshandeh

**Affiliations:** 1 Avicenna Research Institute, Dental Research Center, Oral and maxillofacial Surgery Division, Mashhad University of Medical Sciences, Mashhad, Iran; 2 Cancer Research Center, Omid Hospital, Mashhad University of Medical Sciences, Mashhad, Iran; 3 Department of Oncology, Omid Hospital, Mashhad University of Medical Sciences, Mashhad, Iran

## Abstract

**Background:**

Inadequate epidemiologic investigations of the paranasal sinuses malignancies prompted this retrospective study with special emphasis on a major group of 111 tumors.

**Material and Methods:**

Clinical records of 111 patients with histologically confirmed malignant tumors of the paranasal sinuses were investigated retrospectively from April 2000 to January 2012. Collection of data included demographic information, clinical manifestations, treatment plans, and histopathology of the tumor.

**Results:**

There were 69 (62.16%) male and 42 (37.83%) female patients (male-to-female ratio of 1.6:1), with a median age of 49±12.2 years (range 21 to 88 years). A high level of occurrence was noticed in the fifth (26.3%) decade of life. The most frequent histological types were squamous cell carcinoma (43.5%) and adenoid cystic carcinoma (19%). Among clinical manifestations, nasal obstruction was the most frequent followed by diplopia, and facial swelling. Fifty three patients (47.74%) were treated with combined approach of surgery and radiation therapy.

**Conclusions:**

Paranasal sinuses malignancies are rare conditions with nonspecific symptoms which make early diagnosis of the lesions more challenging. The optimal therapeutic protocol for patients suffering from these tumors is still a somewhat controversial entity and requires further studies.

**Key words:**Paranasal sinuses, malignancy, surgery,radiotherapy.

## Introduction

Malignant tumors of the paranasal sinuses are rare but serious complications in adults (1). There are different factors making diagnosis of these tumors a major challenge including different histological variations, limited anatomic access, and nonspecific clinical manifestations (2,3).

Air-filled structure of sinuses makes these tumors locally aggressive. Malignancies of the paranasal sinuses are usually presented by extension to the adjacent critical organs, which impairs the survival of the patients (4). Patients may present with nonspecific symptoms such as sinusitis, bleeding of the nose or more advanced symptoms when the lesion reaches the skull base or the orbit (5). Invasion of these tumors to skull base, optic nerve and brain stem makes treatment and rehabilitation of patients more complicated (6).

The therapeutic approach for malignancies of the paranasal sinuses is multidisciplinary and consists of surgical and radiological interventions. Due to the rarity and diversity of these tumors and insufficient number of clinical studies, there is no consensus about therapeutic approach for the treatment of tumors of the paranasal sinuses (3).

Optimal imaging is needed for determining the origin and distribution pattern of the tumor (3,7). Surgical treatment protocols have evolved from invasive craniofacial resection surgery to more conservative endoscopic sinus surgery, in order to lower morbidity rate and improve treatment outcome (8,9). Several radiotherapy techniques have been used in combination with surgical procedures to treat patients. Radiotherapy and chemotherapy are considered as an alternate treatment modalities for the patients who are not indicated for the surgical procedure (4).

Inadequate epidemiologic investigations of the paranasal sinus malignancies prompted this retrospective study with special emphasis on the major group of 111 tumors.

## Material and Methods

One hundred and eleven patients diagnosed with malignant tumors of the paranasal sinuses that were treated and followed up at an oncology institute between April 2000 and January 2012 were selected for the present study.

The Ethics Committee of the Medical school approved the study protocol. The diagnosis of the paranasal sinuses malignancies was histologically confirmed in all patients. Exclusion criteria were presence of benign condition, secondary involvement of the paranasal sinuses by tumors of distant area and nasopharyngeal lesions.

Tumor origin and distribution pattern were recognized by clinical and radiographic examinations. Records of the applied therapeutic modalities were made at oncology center. We used the institution surgical and discharge registries to evaluate the cases and extract information about the variables of interest including diagnosis, histological results, treatment approaches and recurrences. The data were analyzed using SPSS 15 software (SPSS, version 15, Chicago, U.S).

Results

There were 69 (62.16%) male and 42 (37.83%) female patients (male-to-female ratio of 1.6:1), with a median age of 49±12.2 years (range 21 to 88 years).

The most frequent reported symptoms at the time of diagnosis were nasal obstruction, diplopia, facial swelling, epistaxis, proptosis, headache, mass in the nose, and auditory disturbance ( Table 1).

**Table 1:**
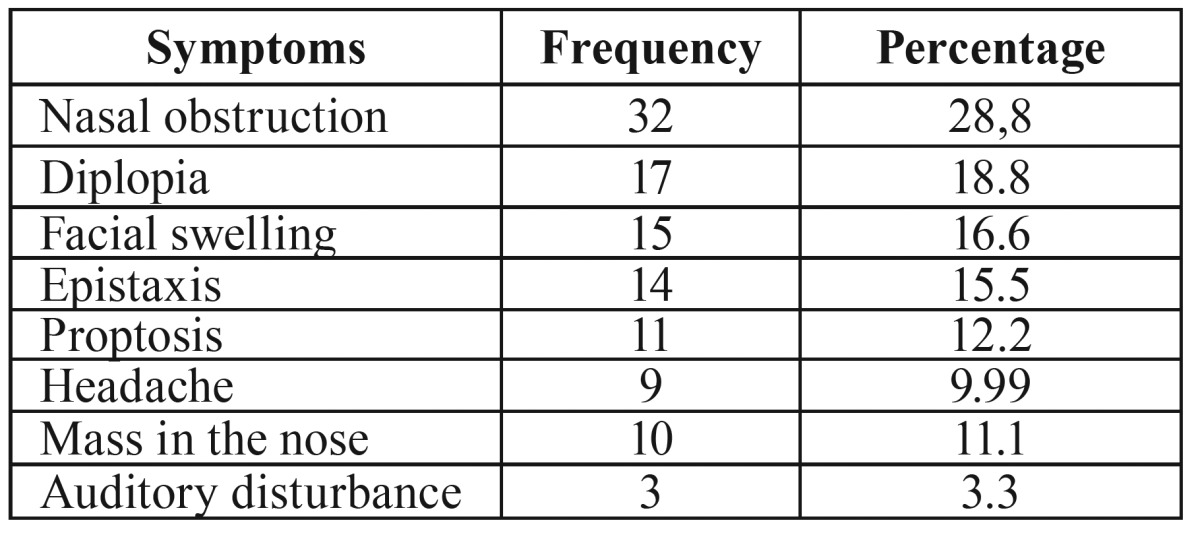
The most frequent clinical manifestations at presentation.

**Figure 1:**
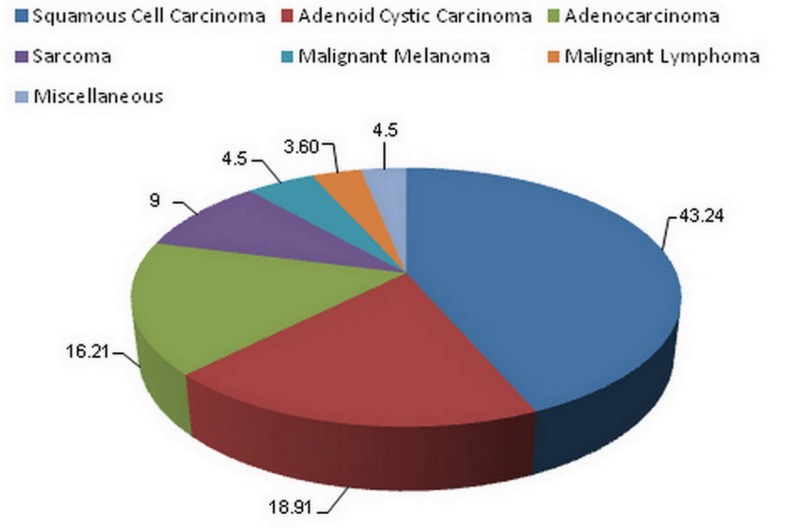
Histological types of tumors.

A high level of occurrence was noticed in the fifth (26.3%), sixth (22.7%) and seventh (18%) decades of life, in descending order. Tumor histological diagnosis revealed that squamous cell carcinoma (SCC) was the most frequent malignant tumor (n = 48) followed by adenoid cystic carcinoma (n = 21), adenocarcinoma (n = 18), sarcoma (n = 10), malignant melanoma (n = 5), malignant lymphoma (n = 4), and other types of carcinoma (n=5) (Fig. 1).

Aggressive local extension and destruction of adjacent structures were observed in most patients. At the time of referral, 62 (55.85%) cases presented with orbit involvement. Principle clinical manifestation and ophthalmic symptom in different histological subtypes are presented in  Table 2. Distant metastasis was noticed in 30 (27%) patients at the time of diagnosis of the paranasal lesion.

**Table 2:**
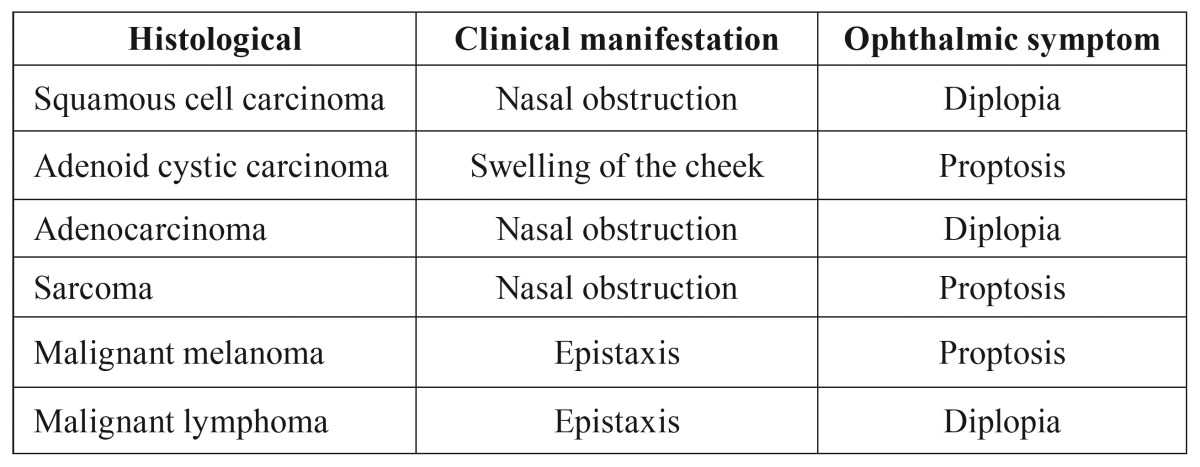
Principle clinical manifestation and ophthalmic symptom in different histological subtypes.

The anatomic location and extent of tumors varied considerably. Tumors of the paranasal sinuses originated from the maxillary sinus in 60 patients, ethmoid sinus in 39 patients, sphenoid sinus in 11 patients, and frontal sinus in 1 patient. In 25% of cases, the lesion involved more than one sinus ( Table 3).

**Table 3:**
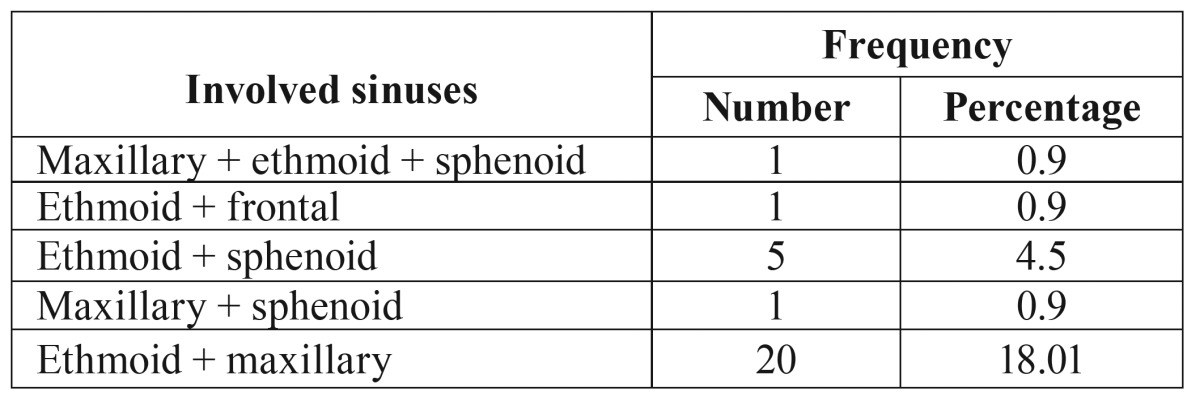
In advanced cases the lesion involves more than one sinus.

None of the patients received palliative treatment. In the present study, 53 patients were treated with combined approach of surgery and radiation therapy, and 10 with surgery alone. Nineteen patients were managed with chemoradiation therapy, and 11 with radiotherapy alone. A combination of surgery and chemotherapy was performed in 18 patients.

Endoscopic sinus surgery was the surgical approach for treatment of the lesions in 17 cases, whereas the remaining 94 patients were treated with conventional resection.

Recurrences were noticed in 61 (55%) patients as local; in 14 (13%) patients as local and distant metastases; in 6 (6%) patients as local and regional recurrences; in 2 (2%) patients as local, regional, and distant metastasis; in 14 (13%) patients as distant metastasis alone; and twelve (11%) patients as regional recurrences alone.

## Discussion

Cancer of the paranasal sinuses are rare, yet challenging, condition with an unknown etiology (1). Epidemiologic investigations provide further information on the prevalence and symptoms associated with these conditions, which lead to early diagnosis or referral of the patient for the treatment. To the best of our knowledge, this group of 111 patients with malignancies of the paranasal sinuses, treated within 12-year period, represents the largest reported series in an Iranian population.

In the current study, median age of patients was 49±12.2 with male to female ratio 1.6:1. As we found in our study, the predominance of men was obvious in previous works (10,11). The majority of neoplasms occurred in the fifth decade of life which are in support with findings in previous studies (2,3).

According to the literature, carcinomas constitute more than 65% of malignant neoplasms of the paranasal sinuses (12,13). In concordance with other reports, the most prevalent malignant tumors in this study were squamous cell carcinoma, adenoid cystic carcinoma, and adenocarcinoma. (1,12) However; Zbaren *et al.* (14) reported epidermoid carcinomas (56%) and adenocarcinomas (14%) as the most common neoplasms of 216 cases. A systematic review by Dulguerov *et al.* (15) evaluated sinonasal tumors over the period of 38 years and found SCC as the most common tumor, which is in support of our results.

Our results are in good agreement with the previous studies where the most frequent primary site of tumors was maxillary sinus (87%) followed by ethmoid sinus (56%) (12,13). Malignant tumors of the frontal sinus are very rare and mainly documented as case reports in literature (16). In our study, frontal sinus malignancy was found only in one case.

In the current study, most common symptoms were nasal obstruction, diplopia, and facial swelling. This was in contrast to the study of Fasunla *et al.* (17) who noticed epistaxis and swelling of the face as the first and fourth most common clinical symptoms. The discrepancy between our results and previous studies may be due to the number of patients evaluated in different studies, ignoring nonspecific symptoms and late diagnosis of the lesions.

There is a gap between the onset of symptoms and final diagnosis of the disease in most cases due to nonspecific manifestations of the paranasal malignancies, which misleads patients to receive treatment in an appropriate time (18,19).

Epistaxis and diplopia more than any other symptoms may provoke patients for further medical consultation due to the fear of bleeding and visual impairment (19). In the present study, diplopia was the most frequent ophthalmic symptom in patients with SCC, adenocarcinoma, and malignant lymphoma. Epistaxis was the chief complaint in 4 out of 5 patients with malignant melanoma. In our study, three patients (3.3 %) suffered from auditory disturbance, which was rarely reported as clinical symptom in previous works (6,10).

Paranasal sinuses malignancies vary in behavior according to anatomical location and histology. Local recurrence was the dominant treatment failure in our patients. In addition, 20 (18%) patients were recorded with regional recurrences and 30 (27%) patients had a distant metastasis, which are in agreement with the previous studies (1,10).

The optimal therapeutic protocol for patients suffering from cancers of the paranasal sinuses is still a somewhat controversial entity (20,21). Since most patients are diagnosed at advanced stages, multidisciplinary approach is preferred to treat the disease. Because of the critical position of the sinuses adjacent to brain and orbit and their bony structure, definitive radiotherapy has not been considered as the only therapeutic approach (1,20). On the other hand, craniofacial resection of malignant tumors has high morbidity and involves further treatments to reconstruct the surgical defect. Many clinicians consider combination of surgery and radiation therapy as a treatment modality for treating aggressive lesions (10,22). Majority of patients in this case series were treated with surgery combined with radiation therapy.

Endoscopic or craniofacial resection are the main surgical methods applied for optimal treatment of patients (1,23). A systematic review of the literature by Higgins *et al.* (24) on the results of craniofacial versus endoscopic resection of sinonasal malignancies revealed that endoscopic management of sinonasal cancers appears to be a promising approach in treatment of low-staged esthesioneuroblastomas and adenocarcinomas. In the present study, 85% of patients were treated with craniofacial resection.

In order to decrease local recurrence rate of aggressive tumors, craniofacial resection with modern radiation therapy techniques were suggested by some studies (1). It is also noteworthy that concomitant chemoradiation therapy is recommended to enhance therapeutic outcome in patients with aggressive local craniofacial malignant tumors (25). Further randomized controlled clinical trials with an adequate sample size are needed to standardize the treatment approach for the treatment of this condition.

In conclusion, sinonasal malignancies are rare group of cancers with squamous cell carcinoma and adenoid cystic carcinoma as the most frequent histological types. Local recurrence was the main common cause of failure followed by distant metastasis and regional relapse of the lesions. The optimal therapeutic protocol for patients suffering from these tumors is still a somewhat controversial entity. However; combined approach including surgery and radiation therapy of patients is recommended. Further investigations are required for more precise therapeutic protocol.
